# Video-Based Plastic Bag Grabbing Action Recognition: A New Video Dataset and a Comparative Study of Baseline Models

**DOI:** 10.3390/s25010255

**Published:** 2025-01-04

**Authors:** Pei Jing Low, Bo Yan Ng, Nur Insyirah Mahzan, Jing Tian, Cheung-Chi Leung

**Affiliations:** NUS-ISS, National University of Singapore, Singapore 119615, Singapore; e1111871@u.nus.edu (P.J.L.); e0052887@u.nus.edu (B.Y.N.); e1112628@u.nus.edu (N.I.M.); cc.leung@nus.edu.sg (C.-C.L.)

**Keywords:** plastic bag grabbing, self-checkout, action video recognition

## Abstract

Recognizing the action of plastic bag taking from CCTV video footage represents a highly specialized and niche challenge within the broader domain of action video classification. To address this challenge, our paper introduces a novel benchmark video dataset specifically curated for the task of identifying the action of grabbing a plastic bag. Additionally, we propose and evaluate three distinct baseline approaches. The first approach employs a combination of handcrafted feature extraction techniques and a sequential classification model to analyze motion and object-related features. The second approach leverages a multiple-frame *convolutional neural network* (CNN) to exploit temporal and spatial patterns in the video data. The third approach explores a 3D CNN-based deep learning model, which is capable of processing video data as volumetric inputs. To assess the performance of these methods, we conduct a comprehensive comparative study, demonstrating the strengths and limitations of each approach within this specialized domain.

## 1. Introduction

Over the years, environmental issues caused by the use of disposable plastic bags have been topics for sustainability and climate change discussions [1]. Governments across the globe are starting to recognize the severity of the impact and have implemented measures to curb the use of disposable plastic bags. For example, the United Nations invited a transformation of the world by announcing the 2030 Agenda for Sustainable Development [2,3]. Carrier bags, being light, inexpensive, and convenient, are excessively used in the daily lives of humanity to carry groceries and goods from shops to home [4].

In an effort to combat plastic pollution and promote environmental sustainability, Singapore has introduced a plastic bag surcharge policy in retail and supermarkets. Starting July 2023, major supermarket chains are required to charge a minimum of five cents for each plastic bag provided to customers [5]. This initiative aims to reduce the excessive use of single-use plastic bags, encouraging consumers to adopt reusable alternatives. By implementing this policy, Singapore joins a global movement towards reducing plastic waste and protecting the environment, fostering a culture of sustainability among its citizens and contributing to long-term ecological preservation. To manage such payments, supermarkets provided an additional payment option or bar code to scan for the “purchase“ of plastic bags or used plastic bag dispensers at the self-checkout counters [6].

It is challenging to monitor the usage of plastic bags in self-checkout areas. Current enforcement methods to ensure plastic bag payments rely heavily on manpower to monitor the use of plastic bags or adopt an honor system in which supermarket operators trust that customers will pay for the plastic bags they use [7]. In addition, owing to the lack of space in self-checkout areas, only standard-size bags can be supplied to supermarkets with plastic bag dispensers [8].

Computer vision-based solutions hold significant potential to automate the monitoring of plastic bag usage in self-checkout areas in supermarkets. Firstly, they provide a passive and non-intrusive method for monitoring, ensuring that the shopping experience is not disrupted. Secondly, the existing CCTV infrastructure, commonly present in self-checkout areas, can be used to implement these vision-based systems without requiring substantial additional investments [9]. Using advanced image processing and machine learning algorithms, these systems can accurately detect and track plastic bag usage, ensuring compliance with store policies and reducing the environmental impact of excessive plastic use. Motivated by this, this paper proposes to take advantage of deep learning-based video analytics methods to develop an action recognition system applied to CCTV footage to detect the action of taking plastic bags. Automation of monitoring would not only detect but would also discourage such unwanted actions as taking plastic bags without payment [10]. In general, this system offers a scalable solution for detecting non-compliant customers and eliminates the need for additional manpower to monitor the use of plastic bags provided at self-checkout counters [11].

**Problem formulation in this paper**: Our study formulates the task as a video classification problem because it focuses on establishing a foundational method for automatically detecting the presence of plastic bag-taking actions in controlled scenarios. At this stage, the primary goal is to determine whether or not a bag-taking event occurs within a video segment, serving as a proof-of-concept for implementing computer vision models in self-checkout environments. This binary classification framework allows us to refine our dataset, evaluate model performance, and ensure that the system can reliably identify bag-taking actions. We recognize that real-world deployment in supermarket scenarios would indeed involve more requirements, such as handling untrimmed videos that capture the entire checkout process and counting the number of bags taken. These tasks are not the focus of the current paper. Instead, the proposed video classification system is a starting point. Future extensions can build upon this groundwork by integrating models that can process untrimmed sequences and incorporating counting mechanisms.

**Contributions of this paper**:

- A benchmark bag-grabbing video dataset is established. We collected 989 video clips for two categories, whereby positive clips contain the action of taking the plastic bag provided, while negative clips reflect other actions besides the said action. To the best of our knowledge, this is the first dataset for this problem, as reviewed in Table 1.
- The plastic bag grabbing is a niche action recognition problem. For that, we designed three baseline approaches, including (i) a hand-crafted feature extraction plus a sequential classification model, (ii) a multiple-frame *convolutional neural network* (CNN)-based action recognition model, and (iii) a 3D CNN-based deep learning-based action recognition model. We provided a comparative study by evaluating these baseline approaches using our benchmark dataset.

The rest of the paper is structured as follows: Section 2 provides a brief review of existing related datasets, including skeleton-based hand action recognition and video-based hand action recognition. The data collection and data preparation process for the benchmark bag grabbing video dataset is described in Section 3. Section 4 presents the three different CNN-based baseline approaches explored in this study, while Section 5 evaluates the three techniques in extensive experiments. Section 6 discusses the limitations and potential impacts of our work. Lastly, Section 7 concludes the paper.

## 2. Related Works

This paper investigates the problem of plastic bag grasping. It requires addressing the unique characteristics of plastic bags and the dynamics of interaction between the bag and the human hand. To our knowledge, no prior work in the literature has specifically addressed this exact problem. Therefore, this section provides a review of existing relevant research, focusing on studies related to plastic bag object detection [12,13,14], human–object interaction [15,16,17,18], and video classification [19,20,21,22,23,24,25,26].

**Plastic bag object detection**: As summarized in Table 1, no existing publicly available dataset that has action on humans withdrawing plastic bags is found. However, there are available datasets related to plastic bag detection and human interaction with plastic bag. For plastic bag detection, there are existing datasets at Roboflow [12,13] and Kaggle [14], which mostly consisted of used plastic bags rather than new plastic bags.

**Human–object interaction**: There are a few studies [27,28,29,30,31] on hand action recognition, including machine learning-based approaches, sensor technologies, and evaluation metrics. They address challenges in gesture recognition systems for human–computer interaction, emphasizing the importance of accurate hand tracking and robust feature extraction. However, despite the richness of research in general hand action recognition, this paper focuses specifically on hand-bag interaction actions. By narrowing the scope to this specific interaction, this study seeks to address the unique challenges and requirements associated with recognizing actions in practical scenarios, such as those encountered in supermarket checkout environments. Motivated by this observation, only research works related to hand–bag interaction action are discussed in Table 1 in this paper.

For example, an existing dataset such as UCF101 [32] contains 13,320 video clips, which are classified into 101 categories, which are further split into 5 types of action, including human–object interaction. Another similar dataset, HMDB51 [33], consists of 6766 video clips from 51 action categories. A hand washing video classification is studied in [34], and a yoga pose classification is studied in [35]. These established action recognition datasets are focusing on human body action and are not specific to plastic bag interaction. Although hand action recognition is studied using general gesture action datasets [15,16,17], general action recognition models cannot be applied to plastic bag grasping. There is also a dataset related to humans’ actions on plastic bags, in particular knotting plastic bags [18], but the action is very different from the act of taking plastic bags.

**Video classification**: Action video understanding is a rapidly evolving field that addresses the automatic interpretation and classification of human activities captured in videos. Broadly, this research area can be viewed from three key perspectives: recognition objective, backbone modeling, and deployment strategies.

- Recognition objective: Action video understanding commonly centers on two related but distinct tasks, including action recognition and action detection. Action recognition focuses on classifying an entire video clip based on the action it contains [30]. This can be further divided into trimmed and untrimmed scenarios. In trimmed action recognition, the action extends across the entire duration of the video. In contrast, untrimmed action recognition deals with videos that include additional irrelevant segments before or after the target action. On the other hand, temporal action detection aims to identify not only the type of action but also its precise start and end times within an untrimmed video [19].
- Backbone modeling: The second perspective focuses on the choice of backbone models [20]. Popular approaches have leveraged CNNs to extract spatiotemporal features. Such methods typically stack 2D or 3D convolutions to capture both spatial patterns within individual frames and temporal patterns across frames. Recently, transformer-based architectures have emerged as a compelling alternative. These models first tokenize video frames into a sequence of embeddings and then use multi-head self-attention layers to capture long-range dependencies. The final classification layers map these embeddings to action categories. Representative transformer-based models include a pure Transformer architecture that models video data as a sequence of spatiotemporal tokens [21], a factorized self-attention mechanism to independently process temporal and spatial dimensions [22], a multiscale vision transformer [23], and a video swin transformer that adapts the swin transformer’s shifted windowing strategy to the video domain [24].
- Deployment strategies: There are two types of deployments of action understanding models, including supervised classification (the focus of this paper’s study) and zero-shot classification. In the supervised setting, models are trained on labeled datasets and then applied to testing examples. However, zero-shot classification has emerged to address scenarios where no training examples for a given action class are available. Leveraging auxiliary modalities (e.g., text embeddings), zero-shot models can classify new, unseen action categories without additional supervision [25,26].

## 3. Dataset

### 3.1. Data Collection and Organization

As the payment of plastic bag use was only mandated last year [5], there are no annotated datasets available that differentiate between the action of taking and not taking plastic bags. Thus, a new benchmark bag-grabbing video dataset is collected in this paper.

**Figure 1:**
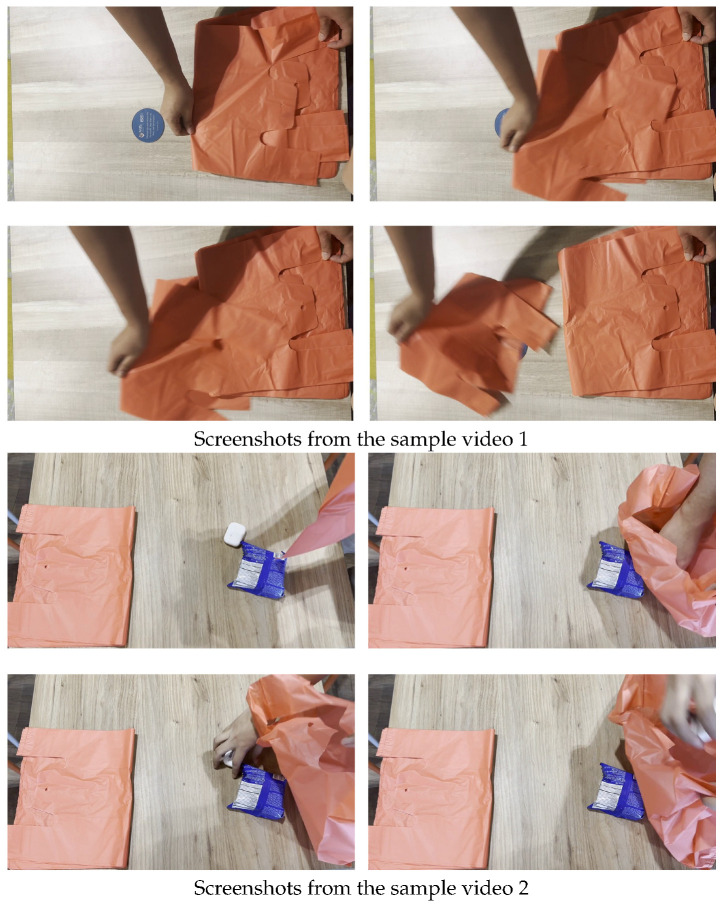
Screenshot examples of action videos collected in this paper.

The dataset is collected as follows. Simulated checkout processes are recorded at 30 frames per second (fps) with the following conditions:

- Each process began with an empty counter, followed by placing items on the counter and packing items into plastic bags. The video either recorded the customer taking the plastic bags from the counter or introducing their own bag. Lastly, both items and bags are removed from the counter.
- A variety of “plastic bag taking” actions are represented, which included pinching and grabbing the bag, as well as the use of both left and right hands, as illustrated in Figure 1.
- Only red-coloured bags are used to represent the plastic bags at the self-checkout counter. This is consistent with the observation that a store typically provides a standard design of plastic bags.
- The customer may touch the bag at the self-checkout counter without taking the plastic bags in the end.

**Figure 2:**
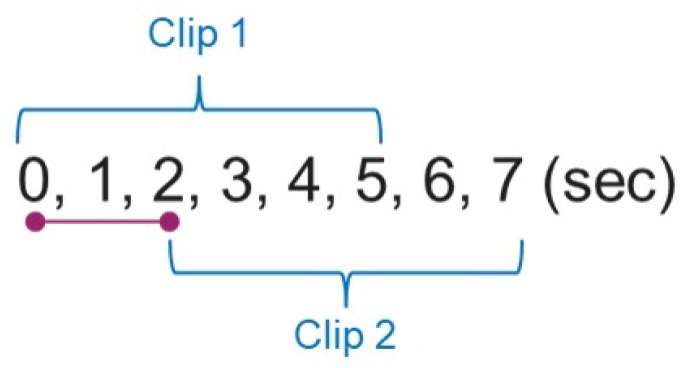
An illustration of how a video sequence is cropped into various clips used in our dataset.

The videos are subsequently segmented into clips of 5 s and repeated after 2 s delay (3 s overlapping from the clip to clip), as shown in Figure 2. The clips are manually labelled as positive and negative according to the action reflected in the clips. There are 989 clips in total; 318 clips are labelled positive and the remaining 671 clips are labelled negative.

### 3.2. Limitation of This Dataset

The collected video dataset exhibits several key limitations that could affect its applicability to broader real-world conditions, although it is useful for modeling self-checkout scenarios.

First, there is a lack of diversity in both objects and actions. It only includes one type of object, which is a red plastic bag. It narrowly represents the range of items, colors, shapes, and textures that might be encountered in actual checkout situations. Second, the absence of other objects besides the red plastic bag reduces the complexity of the visual patterns. Without this diversity, any trained video recognition model might overfit to the limited conditions and struggle to perform robustly with new and unseen scenarios.

## 4. Three Baseline Approaches and a Comparative Study

General action recognition models face significant limitations when applied to plastic bag grasping due to its unique challenges. Firstly, plastic bags are highly deformable, making it difficult to predict their shape and motion compared to rigid objects. Secondly, the interaction between the plastic bag and human fingers requires precise grip adaptation, which involves complex contact points and pressure adjustments that general models are not designed to manage. Thirdly, the variable transparency and reflectivity of plastic bags interfere with visual feature extraction, causing issues in accurately capturing and interpreting the hand’s movements and the bag’s responses.

### 4.1. Selection of Three Baseline Approaches

**Figure 3:**
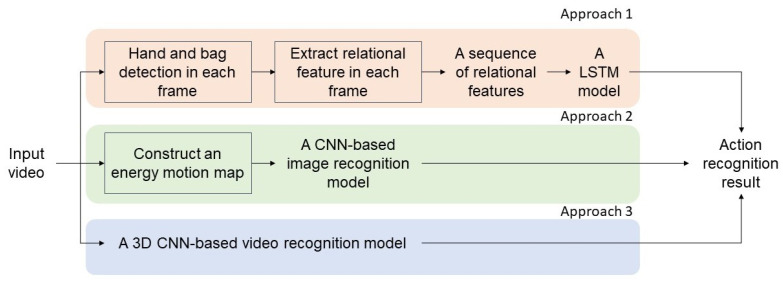
A conceptual overview of three baseline approaches in our study.

Considering the aforementioned challenges and rapid innovation in action video understanding, this study focuses on CNN-based, supervised video classification methods, highlighting how various approaches capture and utilize spatiotemporal relationships in video sequences. To illustrate and compare different strategies for extracting spatiotemporal features, we have selected three representative baseline approaches. Two of these approaches rely on handcrafted feature extraction, following traditional computer vision pipelines that emphasize explicit design and engineering of features. Such methods often offer interpretability and can serve as robust baselines, though they may be limited by the quality and scope of the handcrafted features themselves. In contrast, the third approach adopts a data-driven perspective, allowing a CNN-based model to learn the most relevant spatiotemporal patterns directly from the raw video input. This approach demonstrates the power of end-to-end learning, as it can uncover subtle or complex patterns that might be missed by human-designed features. A conceptual overview of these three baseline approaches is illustrated in Figure 3.

### 4.2. Approach 1: Hand-Crafted Features + LSTM

The basic idea of the first approach is to utilize the relationship between hand keypoints and a plastic bag’s bounding box, then further exploit these features as inputs for a *long short-term memory* (LSTM) model. In this approach, Mediapipe [36] is used to extract hand keypoints, yielding 126 coordinates per frame. Then, a pre-trained bag detector model, YOLOv5, which is trained on a custom plastic bag dataset, is applied to detect the bags. The relational features are extracted by measuring distances between hand keypoints and the bounding box corners. These features are combined in two different options, including early fusion and late fusion, to be used for the LSTM model to perform action classification.

**Figure 4:**
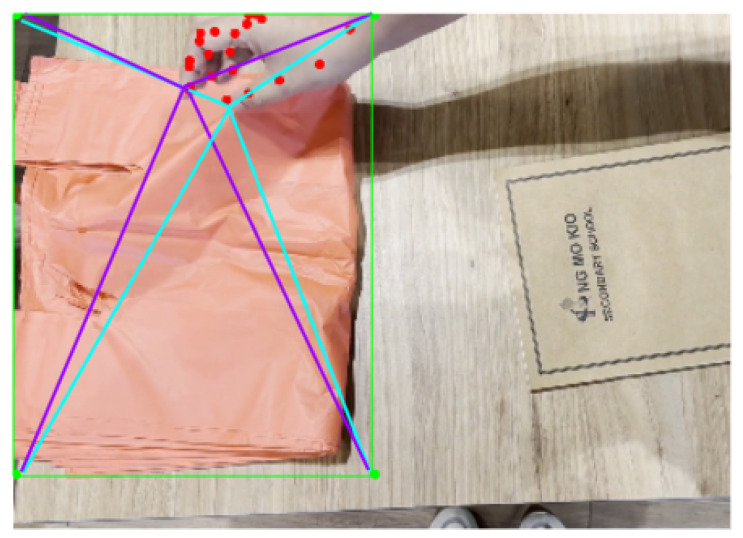
An illustration of relational feature extraction by calculating the distances between the index and thumb of the hand and the four corners of the bounding box of the detected plastic bag.

**Figure 5:**
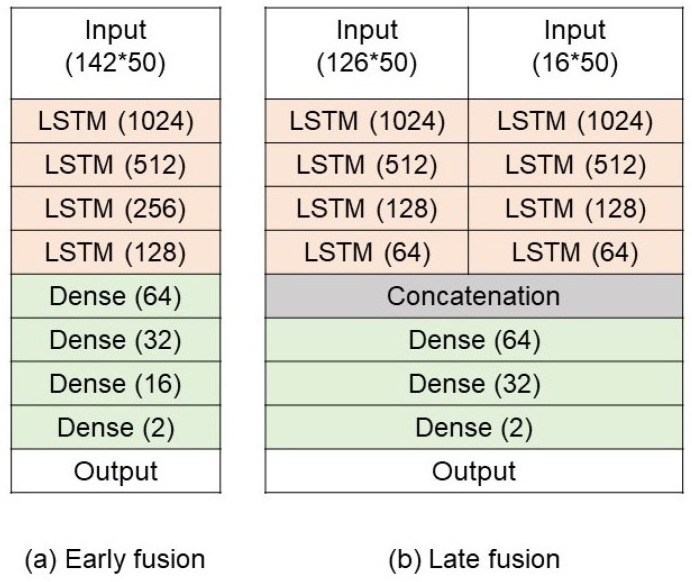
A conceptual overview of two fusion strategies used in our Approach 1, including (**a**) early fusion and (**b**) late fusion.

The details of this approach are described as follows:

- **Hand landmark detection.** A pre-trained model, Mediapipe Hands [36], is used for hand landmark detection. The model is capable of localising 21 hand landmarks, consisting of coordinates and depth information for each hand. This gives rise to 126 location values when both hands are detected in the frame.
- **Plastic bag detection.** A custom-trained Yolov5s model [37] for plastic bag detection is trained using the plastic bag dataset [14]. The rationale for only using this dataset is because this dataset mainly consists of plastic bags, which is relevant to this study.
- **Relational feature extraction.** The relation feature is then extracted by calculating the distances between the index and thumb of the hand and the four corners of the bounding box of the detected plastic bag, as illustrated in Figure 4. The relationship information is extracted from each frame, and the sequence of such features is used as inputs for the next classification model. This gives rise to 16 values in each frame.
- **Action classification.** Two variations are explored for the technique of using the hand-crafted features and LSTM, mainly early fusion and late fusion, as presented in Figure 5. The key difference between early and late fusion is the inputs for the LTSM model. To be more specific, early fusion uses both hand keypoints and relationships between the hand and plastic bag within the same LSTM model, while there are separate LSTM models for hand keypoints and relationships between the hand and plastic bag in late fusion. In early fusion, the hand keypoints and relational features are combined into a single input vector before being fed into a single LSTM model. This means that the model learns an integrated representation of hand pose and the spatial relationship to the plastic bag from the very beginning. In contrast, late fusion maintains two separate independent LSTM models, including one for the hand keypoints and another for the relational features. Only after both models have processed their individual feature streams do their outputs get merged. This approach allows each LSTM to specialize in a particular feature domain and reduces the complexity of the data representation.

### 4.3. Approach 2: Energy Motion Image + Image Classification

The objective of the second baseline approach is to exploit a motion image extraction from the input action video followed by an image classification. For that, the *energy motion image* (EMI) method is applied by generating an image representation of motion via accumulating differences between consecutive video frames, thus highlighting the energy or movement in specific regions of the video. Firstly, it applies Mediapipe to attain hand keypoints on each video frame in the clip, and the lines connecting the hand keypoints are thickened to mimic the hand skeletal structure. Subsequently, the EMI image is generated by performing a weighted overlay of the hand keypoints across frames, whereby the hand keypoints of the latest frame contribute more to the final EMI. These EMI images are used as inputs for a single image classification model (i.e., a ResNet model).

There are a few different approaches to assigning weights that place increasing emphasis on later frames, including linear weighting and quadratic weighting. Given a list of frames denoted as $x_{1},x_{2},\ldots ,x_{n}$, they can be combined to obtain a final EMI image $y=\sum_{i=1}^{n}w_{i}x_{i}$, where $w_{i}$ is the weighting factor for the *i*-th frame defined as

(1) $$\begin{array}{ccc} \mathrm{Linear}\;\mathrm{weighting}:\;w_{i} & = & \frac{i}{\sum_{k=1}^{n}k}, \end{array}$$

(2) $$\begin{array}{ccc} \mathrm{Quadratic}\;\mathrm{weighting}:\;w_{i} & = & \frac{i^{2}}{\sum_{k=1}^{n}k^{2}}. \end{array}$$

A larger frame index *i* (latest frame) has a larger weighting factor; consequently, the frame has a higher contribution to the calculated EMI image.

The details of this approach are described as follows:

- **Motion energy map.** Hand skeletal structures, if any, are generated using Hand Landmark Detection for every frame in the input video clip. Thus, each EMI comprises a maximum overlay of 150 frames, rendering the latest frame in the video clip to have the greatest weight in the final EMI. These EMIs are used as inputs for an image classification model that determines if the clip reflects the plastic bags taking action.
- **Action classification.** In consideration of the fact that the energy motion image is grayscale, a single-channel image classification model (i.e., ResNet model) is explored. In addition, the EMI image has been downsized to a resolution of 224 × 224 to reduce the number of parameters.

### 4.4. Approach 3: 3D CNN Model

**Figure 6:**
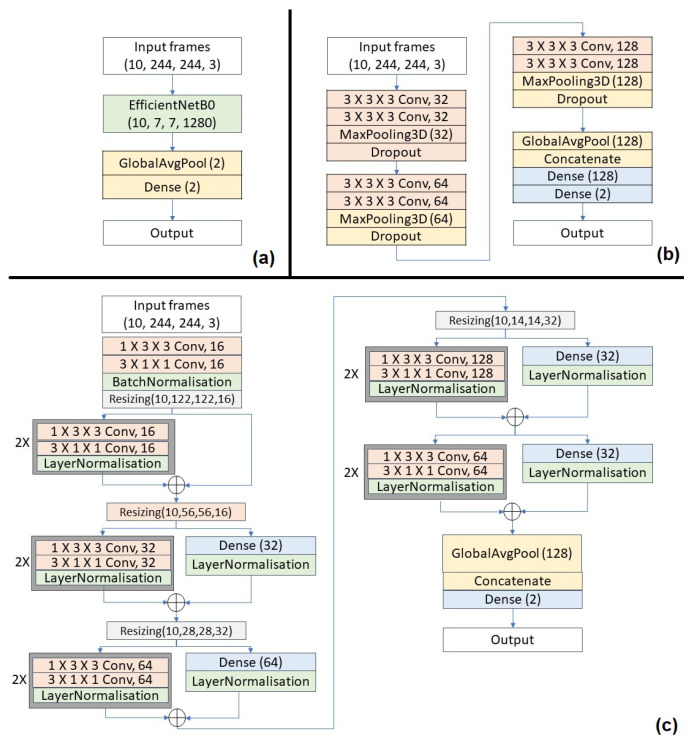
An illustration of three options of the 3D CNN-based approach, including (**a**) a pre-trained EfficientNet model, (**b**) a 3D CNN model, and (**c**) a (2 + 1) D CNN model.

The aim of the third baseline approach is to exploit the 3D CNN model on the input action video sequence directly without prior feature extraction done. This approach combines both feature engineering and classification into a single 3D CNN model that allows the model to deeply learn the key features itself. In addition, three different variants are explored in this approach, as shown in Figure 6.

- **EfficientNet** model [38]. It is re-trained on our benchmark video dataset.
- **3D CNN** model [39]. It generally performs better than 2D networks as it is able to model temporal information better on top of the spatial information that 2D networks capture.
- **(2 + 1)D** ResNet model [40]. The use of (2 + 1)D convolutions over regular 3D convolutions reduces computational complexity by decomposing the spatial and temporal dimensions to reduce parameters. It also prevents overfitting and introduces more non-linearity that allows for a better functional relationship to be modeled.

## 5. Experimental Results

### 5.1. Performance Metrics

The performance evaluation is conducted using four key performance metrics: *accuracy*, *precision*, *recall*, and *F1 score* [41]. Precision evaluates the models’ ability to predict the action of taking plastic bags accurately. Recall evaluates the models’ ability to predict all the actions of taking plastic bags. Accuracy evaluates the models’ ability to differentiate between actions that are not taking plastic bags and actions that are taking plastic bags. The F1 score accounts for both precision and recall and is a better metric for an imbalanced dataset compared to accuracy. In addition, the computational complexity of various approaches is evaluated using the number of trainable parameters, which represents the total number of parameters used for each model.

### 5.2. Implementation Details

The implementation details of three baseline approaches are provided here. All the weights in the model are initialized using HE initialization [42]. Table 2 documents the setting used for the model training. The model is trained for 240 epochs on an Nvidia GTX 2080Ti GPU with the 1.7.1 version of the PyTorch library.

### 5.3. Experimental Results

As seen in Table 3, the late fusion predicts the action more accurately than the early fusion for Approach 1. In addition, Approach 2 is observed to be fairly accurate in detecting the action of taking the plastic bags, with an F1 score of 0.82. Lastly, for Approach 3, it is found that the (2 + 1)D ResNet model can perform the video classification best when compared with the other two video classification modelling techniques.

The computational complexity of various approaches is evaluated on a computer with 16 GB of RAM and a 2.61 GHz CPU processor without a GPU. The detailed comparison is presented in Table 4.

### 5.4. Ablation Study

The first ablation study examines Approach 1 of hand-crafted feature extraction; the effect of the relation features of hand keypoints and the plastic bag is examined (Table 5). This experiment is conducted using the early fusion model. This experiment indicates that the relation features (distances between plastic bag and hand) could be important for the action recognition.

**Figure 7:**
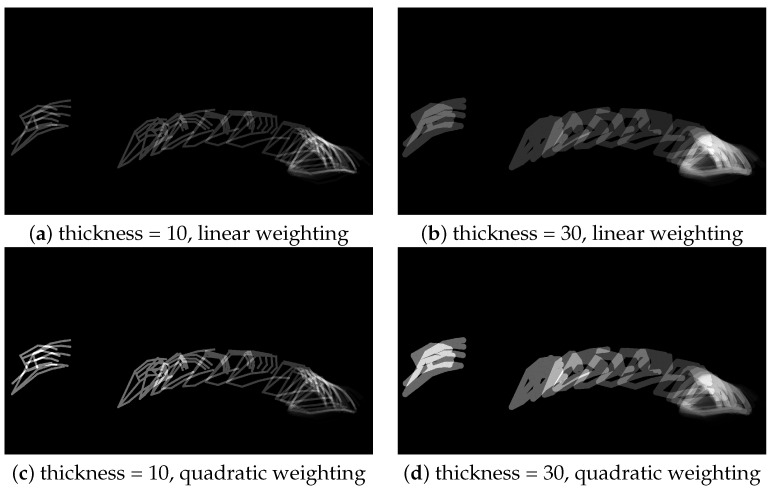
Example of energy motion images generated from overlaying hand keypoints across frames with different skeleton thickness values and weighting methods.

The second ablation study examines Approach 2 of the construction of EMI imaage. There are two hyperparameters used in this approach. The first hyperparameter is the thickness of the hand skeleton structure, which is controlled in OpenCV. It could be adjusted using two options, including a small value of 10 and a large value of 30. The second hyperparameter is the choice of the weighting method that combines multiple frames to obtain a single EMI image. It could be set using either the linear weighting method (1) or the quadratic weighting method (2). The effects of thickness and weighting methods are compared in Table 6 and Figure 7. As shown in Figure 7, increasing the skeleton thickness produces EMI images with larger highlighted regions (see images on the right column in Figure 7). Similarly, using the quadratic weighting method, which assigns higher weights to more recent frames, results in EMI images with enhanced contrast (see images on the bottom row in Figure 7). As seen from Figure 7, a larger skeleton thickness and linear weighting method can achieve better action classification performance than that of a smaller skeleton thickness and quadratic weighting method.

### 5.5. Overall Evaluation

Comparing the results of all three approaches, it is observed that the model performances and number of parameters in Approach 2 and Approach 3 are comparable. However, while the model in Approach 2 is less complex, substantial computational time and resources are required for hand keypoint extraction and image overlay on all 150 frames of each 5-second video clip to generate the EMI image prior to image classification. In contrast, the video classification model selects several frames from the video at predefined intervals as inputs to the model. It is important to note that Approach 2 might overfit to the limited conditions due to the absence of other objects besides the red plastic bag in the dataset.

The performances of the LSTM and ResNet models for Approaches 1 and 2 are dependent on the accuracy of the pre-trained models, including Yolov5 and Mediapipe, for plastic bag detection and hand keypoint detection, respectively, as inputs for LSTM or ResNet models. Due to the nature of the Roboflow plastic bag dataset, the Yolov5 model is observed to be insensitive to the plastic bags with the edges occluded by the customer’s hand, while the coordinates of the plastic bag bounding box of the previous video frames are used as a reference for Approach 1 to mitigate this issue; it would have been preferable to have a more robust plastic bag detection model.

## 6. Limitations and Potential Impacts

We fully acknowledge that our research is focused on the specific task of plastic bag grabbing, particularly within the context of Singapore. This specificity stems from the unique challenges and requirements of this application, such as the distinct (or niche) colors and interactions with plastic bags commonly encountered in Singapore. Despite its niche focus, our work has a significant impact on real-world applications in the retail sector, particularly in cashier-less stores. The ability to accurately detect and classify such specific actions is vital for enhancing operational efficiency and security in these contexts. We believe our released benchmark video dataset and the evaluation of major baseline approaches broaden the relevance of our research beyond academia.

While our study is niche, we see it as a stepping stone for the broader computer vision-based human activity research. The introduction of our dataset and baseline methods can provide valuable insights and a foundation for related tasks, such as fine-grained action recognition and object interaction analysis. Researchers can adapt or extend these methods to other fine-grained action recognition tasks.

Future research in this area can be extended in two significant directions based on the findings of this paper. Firstly, while this work evaluates three CNN-based baselines for action video recognition, future studies could focus on transformer-based methods [20]. Transformers, with their ability to capture long-range dependencies and temporal correlations, hold great promise for action recognition tasks. Secondly, the benchmark dataset presented in this study primarily covers a limited range of scenarios, which might not fully represent the diversity encountered in real-world applications. For instance, the task of recognizing actions related to plastic bag grabbing in supermarket checkout scenarios could benefit from datasets encompassing more diverse and challenging conditions, such as varying backgrounds, lighting conditions, and object types. Expanding datasets to include such out-of-distribution scenarios [43] will be crucial for improving the generalizability and robustness of action recognition systems in practical deployment.

## 7. Conclusions

In this paper, we introduce a novel benchmark dataset specifically designed for the task of recognizing plastic bag grabbing actions from video footage. This dataset addresses a unique and niche challenge in action recognition, capturing the subtleties of human–object interactions involving plastic bags in diverse scenarios. To tackle this task, we have developed three baseline models tailored to this task. They include a handcrafted feature-based model, a multiple-frame CNN-based recognition model, and a 3D deep learning model. Through a detailed comparative evaluation, we highlight the strengths and limitations of each method, providing valuable insights and a robust foundation for advancing research in fine-grained action recognition tasks.

## Figures and Tables

**Table 1:** An overview of the relevant datasets and their comparison with our dataset. Only research works related to hand–bag interaction action are discussed in this paper. Our dataset can be accessed at https://doi.org/10.17632/h3hs3nnvbc.1 accessed on 1 January 2025.

	Ref.	Year	Bag Object	Hand Gesture	Hand Bag Interaction	Available to Public	Remarks
Plastic bag object detection	[12]	2022	√	-	-	√	1 class (plastic bag or not) and 283 images.
	[13]	2022	√	-	-	√	3 classes (Plastic, Paper, and Garbage Bag) and 5000 instances
	[14]	2023	√	-	-	√	6 classes (anorganic, paper bag, shopping bag, smoke, stretch, trashbag) and 400 images
Hand bag interaction	[15]	2016	-	√	-	√	25 types of gestures and 1532 samples
	[16]	2018	-	√	-	√	83 types of gestures and 24,161 samples
	[17]	2019	-	√	-	√	30 types of gestures and 1200 samples
	[18]	2023	√	√	√	-	4 types of bags and 43,000 images
Our dataset		-	√	√	√	√	2 classes and 989 clips

**Table 2:** Implementation details of three baseline models in our study.

	Approach 1		Approach 2	Approach 3		
	Early Fusion	Late Fusion		EfficientNet	3D CNN	(2 + 1)D CNN
Optimizer	Adam	Adam	Adam	Adam	Adam	Adam
Learning rate	0.001	0.001	0.005–0.00005	−	0.0001	0.0001
Batch size	32	32	32	8	8	8
Epoch	30	30	200	20	20	20

**Table 3:** The performance evaluation of three baseline approaches.

	Option	Precision	Recall	F1 Score	Accuracy
	Early Fusion	0.62	0.52	0.57	0.59
Approach 1	Late Fusion	0.85	0.33	0.47	0.62
Approach 2		0.89	0.76	0.82	0.88
	EfficientNet	0.82	0.69	0.76	0.86
Approach 3	3D CNN	0.78	0.88	0.82	0.88
	(2 + 1)D ResNet	0.92	0.91	0.91	0.94

**Table 4:** The computational complexity comparison of three baseline approaches. The YOLO object detection and Mediapipe calculation are excluded from this evaluation.

	Option	Frame Resolution	Inference Frame Rate	# Parameters	FLOPs
Approach 1	Early Fusion	1920×1080	15	8,968,594	17,955,137
	Late Fusion	1920×1080	15	16,056,098	31,927,660
Approach 2		224×224	30	279,778	4,003,459,372
	EfficientNet	224×224	2	4,052,133	8,011,611,411
Approach 3	3D CNN	224×224	2	876,898	59,520,912,012
	(2 + 1)D CNN	224×224	2	586,226	9,160,758,184

**Table 5:** Ablation study of relational features used in Approach 1.

Hand Keypoints	Relational Feature	Precision	Recall	F1 Score	Accuracy
√	-	0.81	0.25	0.38	0.58
√	√	0.62	0.52	0.57	0.59

**Table 6:** Ablation study of hyperparameters used in Approach 2, including skeleton thickness and weighting methods used in the frame combination.

Skeleton Thickness		Frame Combination		Precision	Recall	F1 Score	Accuracy
Small Value	Large Value	Linear Weighting	Quadratic Weighting				
√		√		0.79	0.75	0.77	0.82
	√	√		0.89	0.76	0.82	0.88
√			√	0.81	0.73	0.77	0.82
	√		√	0.83	0.81	0.82	0.77

## Data Availability

Data are contained within the article.
